# Effect of Secondary Aging Conditions on Mechanical Properties and Microstructure of AA7150 Aluminum Alloy

**DOI:** 10.3390/ma18204763

**Published:** 2025-10-17

**Authors:** Fei Chen, Han Wang, Yanan Jiang, Yu Liu, Qiang Zhou, Quanqing Zeng

**Affiliations:** 1 School of Mechanical Engineering and Automation, College of Science and Technology, Ningbo University, Ningbo 315300, China; chenfei@nbu.edu.cn (F.C.); 13656711157@163.com (H.W.); jiangyanan@nbu.edu.cn (Y.J.); 2 College of Mechanical and Electrical Engineering, Central South University, Changsha 410083, China; csuliuyu@csu.edu.cn

**Keywords:** AA7150 alloy, aging treatment, strength, precipitates, microstructure

## Abstract

Al-Zn-Mg-Cu alloys are widely used as heat-treatable ultra-high-strength materials in aerospace structural applications. While conventional single-stage aging enables high strength, advanced performance demands call for precise microstructural control via multi-stage aging. In this study, we employ a combination of scanning transmission electron microscopy (STEM), energy-dispersive X-ray spectroscopy (EDS), and X-ray diffraction (XRD) to investigate the microstructural evolution and its correlation with mechanical properties of AA7150 aluminum alloy subjected to two-step aging treatments, following a 6 h pre-aging at 120 °C. Through atomic-scale STEM imaging along the [110]_Al_ zone axis, we systematically characterize the precipitation behavior of GPII zones, η′ phases, and equilibrium η phases both within the grains and at grain boundaries under varying secondary aging (SA) conditions. Our results reveal that increasing the SA temperature from 140 °C to 180 °C leads to coarsening and reduced number density of intragranular precipitates, while promoting the continuous and coarse precipitation of η phases along grain boundaries, accompanied by a widening of the precipitation-free zone (PFZ). Notably, SA at 160 °C induces the formation of fine, uniformly dispersed nanoscale η′ precipitates in the alloy, as confirmed by XRD phase analysis. Aging at this temperature markedly enhances the mechanical properties, achieving an ultimate tensile strength (UTS) of 613 MPa and a yield strength (YS) of 598 MPa, while presenting an exceptionally broad peak-aging plateau. Owing to this feature, a moderate extension of the SA duration does not reduce strength and can further improve ductility, increasing the elongation (EL) to 14.26%. These results demonstrate a novel two-step heat-treatment strategy that simultaneously achieves ultra-high strength and excellent ductility, highlighting the critical role of advanced electron microscopy in elucidating phase-transformation pathways that inform microstructure-guided alloy design and processing.

## 1. Introduction

AA7150 is a typical representative of the 7xxx series of ultra-high-strength aluminum alloys. It is widely used in primary load-bearing structural components of large airliners, including fuselage frames and wing panels, owing to its excellent specific strength, fatigue resistance, and good formability [1]. With the shift in next-generation aircraft toward lightweight and long-life designs, the performance requirements for aluminum alloy components have become increasingly stringent. In particular, improvements are needed in strength, toughness, and resistance to stress-corrosion cracking. These components must sustain high strength across a wide service temperature range (−50 °C to 150 °C). At the same time, they must also provide superior stress-corrosion resistance to withstand harsh environmental conditions [2,3]. Therefore, considering the strict service demands of large thin-walled components, enhancing their mechanical performance has become an urgent task to meet the requirements of next-generation aerospace engineering.

These alloys are often susceptible to fracture and exhibit reduced ductility in the as-cast condition [4]. To improve their mechanical properties, a combination of heat treatment and hot working has historically been employed [5]. Aging treatment plays a key role in strengthening 7xxx-series aluminum alloys. The standard procedure for artificial peak-aging (T6 treatment) typically involves a heat treatment at 120 °C for 24 h [6]. This produces a uniform distribution of nanoscale precipitates, mainly Guinier–Preston (GP) zones and metastable η′ phases [7,8,9]. However, the 24 h cycle leads to high energy consumption and processing costs, which restrict large-scale industrial application. To shorten the cycle while maintaining strength, many researchers have proposed multi-stage aging as an optimization strategy [10,11,12,13]. In this approach, high-density nucleation of GP zones occurs during low-temperature aging, followed by the precipitation of η′ phases at higher temperatures [14,15]. Compared with conventional aging, multi-stage aging enables more precise regulation of precipitate evolution. It allows simultaneous control over size distribution, composition, crystal structure, morphology, and spatial arrangement of nanoscale particles [16]. These modifications can significantly enhance the mechanical and overall properties of the material. In studies of multi-stage aging processes, numerous researchers have explored the relationship between precipitation phase evolution under varying temperature and time conditions and the mechanical properties of alloys. For instance, Sun et al. [17] showed that multi-stage intermittent aging reduced dislocation density and nearly eliminated residual stress in Al-Zn-Mg-Cu alloys. Yan et al. [18] reported that pre-aging Al-Li alloys at 120 °C for 12 h enhanced diffusion of Cu and Li atoms to {111}_Al_ planes, while retaining vacancies that promoted T1 precipitation. Li et al. [19] employed a reverse aging process to promote dissolution of coarse η phase in 7075 alloy and induce substantial precipitation of nanoscale η′ phase.

As a typical example of multistage aging, the T74 tempering process employs a stepped temperature control strategy: primary aging (T1 stage) is performed at 100–120 °C, followed by secondary aging (T2 stage) at 140–180 °C [20,21]. This staged conditioning mechanism significantly enhances stress corrosion resistance in 7xxx-series (Al–Zn–Mg–Cu) alloys by optimizing precipitate distribution. However, it typically causes a 10–15% reduction in tensile strength compared with the peak-aged T6 condition [22,23,24]. For AA7150, a precipitation-strengthened alloy, the mechanical properties and fracture behavior are governed by the multiscale microstructure formed during aging. Key factors include the size and type of intragranular and grain-boundary precipitates (GBPs), grain orientation, and the width of the precipitation-free zone (PFZ) [25]. Therefore, in view of the aging characteristics of this alloy, it is necessary to further optimize the strength and toughness of AA7150 within the T74 processing framework. At relatively low temperatures, promoting the formation of a high density of uniformly distributed GP zones and early η′ nuclei provides abundant nucleation sites for subsequent aging. By systematically adjusting the secondary-aging temperature and holding time, it becomes possible to suppress the development of coarse continuous GBPs while enhancing the intragranular dispersion of fine precipitates, thereby achieving a synergistic improvement in both strength and ductility.

To investigate the effect of secondary aging treatment on the mechanical properties and microstructure of AA7150 aluminum alloy, various secondary aging processes were examined using tensile testing, scanning transmission electron microscopy (STEM), and X-ray diffraction (XRD). Ultimately, the type and distribution of precipitates in AA7150 were tailored through a secondary aging design, thereby elucidating their influence on the alloy’s mechanical properties.

## 2. Material and Experimental

### 2.1. Materials

**Figure 1:**
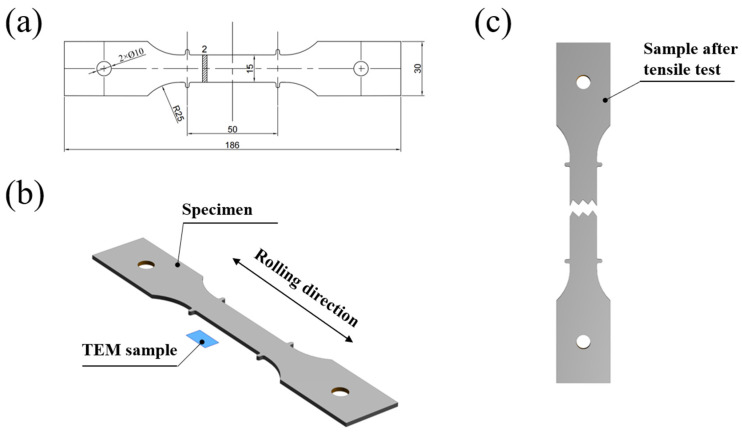
Schematic illustrations of the aged sample and analysis specimens. (**a**) Dimensions of the aged specimen (unit: mm). Schematics of the samples prepared for (**b**) TEM characterization and (**c**) tensile testing.

**Figure 2:**
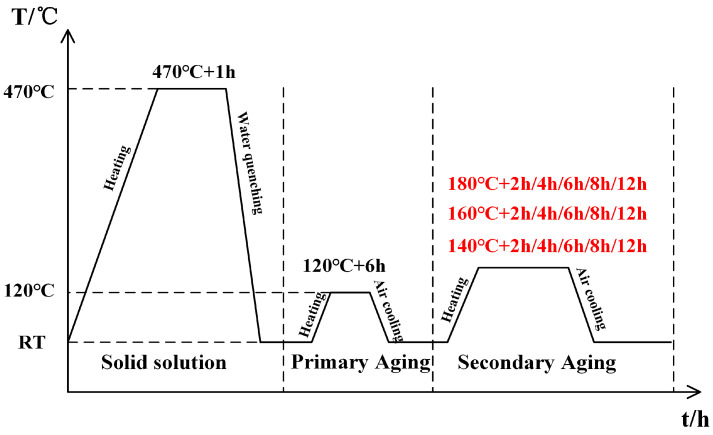
Time/temperature schematic of the whole process chain.

A 35 mm thick commercially available AA7150 aluminum alloy plate was used as the base material for the experiment, with its chemical composition listed in Table 1. Prior to the experiment, the plate required pretreatment, which involved solid solution strengthening at a holding temperature of 470 °C for 60 min, followed by rapid water quenching to room temperature (25 °C) within 5 s. The aluminum alloy plate was then machined into dog-bone-shaped specimens along the rolling direction, with the geometry and dimensions of the specimens illustrated in Figure 1. Before the two-step aging process, the specimens were in the T4 condition, with an ultimate tensile strength (UTS) of 417.6 MPa, yield strength (YS) of 412.3 MPa, and elongation (EL) of 16.3% [26]. All samples were pre-aged uniformly at 120 °C for 6 h before secondary aging. The secondary aging was performed at 140 °C, 160 °C, and 180 °C for 2, 4, 6, 8, and 12 h, followed by air cooling to room temperature. The overall heat treatment procedure is shown in Figure 2. The pre-aging regime of 120 °C for 6 h was selected to establish an optimal microstructural precursor for the subsequent secondary aging. Previous studies [26] have shown that this temperature effectively promotes the formation of a high density of fine metastable η′ nuclei while remaining below the threshold for significant coarsening. A 6 h duration produces a well-developed yet slightly under-aged state, ensuring a uniform distribution of these η′ precipitates. Such a microstructure provides abundant nucleation sites during the second aging stage, refining the final precipitate distribution and enhancing the synergy between strength and ductility. In addition, this pre-aging condition partially consumes supersaturated vacancies, suppressing excessive diffusion and precipitate coarsening at elevated temperatures, thereby offering a broader processing window for optimizing the final mechanical properties.

### 2.2. Mechanical Property Tests

After two-step aging treatments, uniaxial tensile tests were performed on specimens with different secondary aging processes on a 100-kN MTS-Model E45 testing machine (MTS Systems Corporation, Eden Prairie, MN, USA) (equipped with a YYU-12.5-25 electronic extensbbbometer (NCS Testing Technology Co., Ltd., Beijing, China) for precise measurement of pre-yield strain) at room temperature, with a tensile strain rate of 1 × 10^−3^ s^−1^. To ensure data reliability, each test was repeated three times for each set of process parameters. For clarity in presenting the results, the secondary-aged samples were designated using an abbreviated naming convention. For instance, “SA180-2 h” denotes a secondary aging treatment at 180 °C for 2 h, with other samples named following the same pattern.

### 2.3. Microstructure Characterization

Microstructural characterization was conducted using a Thermo-Fisher Talos F200X transmission electron microscope (Thermo Fisher Scientific Inc., Waltham, MA, USA) equipped with a high-angle annular dark-field (HAADF) detector, operating at 200 kV. Initially, the samples were mechanically polished by sequentially grinding their surfaces with abrasive papers of varying grits. Subsequently, the samples were finely polished with a polishing solution to achieve a thickness of approximately 70 μm and then sectioned into thin aluminum discs of about 3 mm in diameter. For STEM analysis, the samples were thinned using a Struers TenuPol-5 double-jet electrolytic polisher (Struers ApS, Ballerup, Denmark) with an electrolyte composed of 30% nitric acid in ethanol, maintained at temperatures below −25 °C and an operating voltage of 15 V.

XRD analysis was employed to examine the evolution of the precipitated phases. The samples were initially mechanically polished to a thickness of 0.2–0.3 mm and then sectioned into rectangular specimens measuring 5 mm in width and 7 mm in length. A Bruker D8 Ultra Front X-ray diffractometer (Bruker AXS, Karlsruhe, Germany), operating at an accelerating voltage of 4 kV and a current of 30 mA, was employed. The scanning mode was set to 2θ, with a scanning angle range of 20° to 90°, and a scanning speed of 2°/min.

## 3. Results

### 3.1. Microstructure of Two-Step Aging Samples

**Figure 3:**
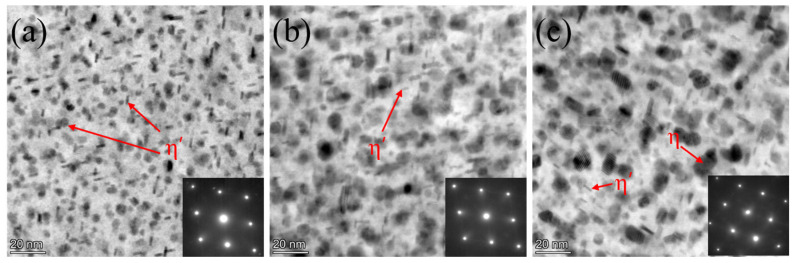
STEM-BF images and SAED spots of intragranular precipitates along the [110]_Al_ direction for secondary peak-aging samples at different temperatures: (**a**) SA140-4 h; (**b**) SA160-6 h; (**c**) SA180-2 h.

Figure 3 shows the microstructural features of Al-Zn-Mg alloys under different peak-aging conditions, observed by scanning transmission electron microscopy (STEM) along the [110]_Al_ zone axis. As shown in Figure 3a, numerous dense and fine needle-like precipitates, ranging in size from 4 to 10 nm, are observed in the bright-field (BF) image. These precipitates are identified as the η′ phase (MgZn_2_), which is the primary strengthening phase in Al-Zn-Mg alloys [27,28]. The η′ phase forms as metastable precipitate during the early stages of aging and is either coherent or semi-coherent with the aluminum matrix, contributing to significant strengthening through dislocation pinning [29,30]. Notably, a portion of near-spherical η′ precipitates (5–8 nm in diameter) was observed, likely resulting from non-uniform nucleation on two mutually perpendicular {111}_Al_ crystallographic planes, a phenomenon influenced by the 35.26° angular difference between the (110)_Al_ and {111}_Al_ planes [26]. This crystallographic constraint results in anisotropic growth kinetics, with the precipitate growing preferentially along the <111>_Al_ direction when viewed along the [110]_Al_ zone axis, resulting in a needle-like morphology in projection. The SA160-6 h sample, with a very fine and diffuse distribution of the strengthened η′ phase (Figure 3b), exhibits spatial homogeneity, which is highly beneficial for the strength of aluminum alloys [31]. In contrast, the precipitates in the SA180-2 h sample begin to coarsen, and a small amount of η phase (MgZn_2_) appears; however, the η′-strengthened phase remains dominant, and the η′ phase is more effective than the η phase for strengthening in Al-Zn-Mg alloys [32].

**Figure 4:**
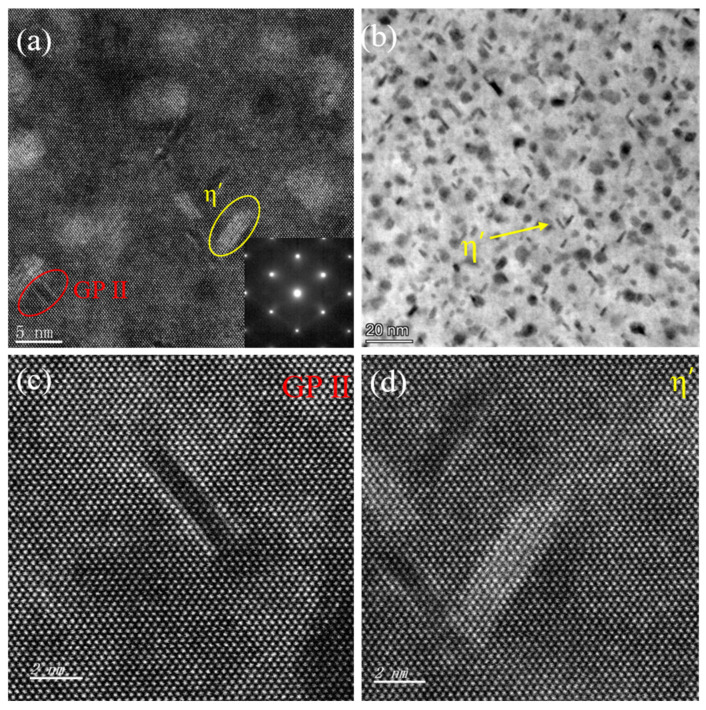
STEM images and SAED spots of the SA160-2 h sample ([110]_Al_ axis): (**a**) High resolution HAADF-STEM images; (**b**) bright-field STEM image; (**c**) magnified view of a GPII zone; (**d**) magnified view of an η′ precipitate.

Figure 4 shows HRTEM and STEM images of the SA160-2 h sample obtained along the [110]_Al_ crystallographic axis, together with the corresponding selected area electron diffraction pattern (SAEDP). Two distinct precipitate morphologies are visible. The GPII zones (circled in red) exhibit a plate-like shape, with a length of 5–10 nm, as shown in the local magnification in Figure 4c. In contrast, the η′ precipitates (circled in yellow) display a rod-like morphology, with an average length of 7.92 nm and a width of 2.2 nm, as shown in Figure 4d. Notably, the GPII zone displays a localized and inhomogeneous distribution, whereas the η′ precipitates are homogeneously distributed throughout the matrix (Figure 4b). A comparison with the SA160-6 h sample (Figure 3b) highlights the dynamic evolution of the precipitate population during prolonged secondary aging. In the SA160-2 h state, residual GPII zones coexist with η′ precipitates, representing an intermediate stage in the transformation of GPII. By contrast, in the SA160-6 h sample, the GPII zones disappear completely and are replaced by a higher density of η′ precipitates. This phase transformation suggests that the nucleation kinetics of the η′ phase surpass the stability of the GPII zone as aging progresses. The marked increase in the density of η′ precipitates in SA160-6 h compared with SA160-2 h indicates that solute redistribution and interfacial energy minimization are promoted by extended thermal exposure.

**Figure 5:**
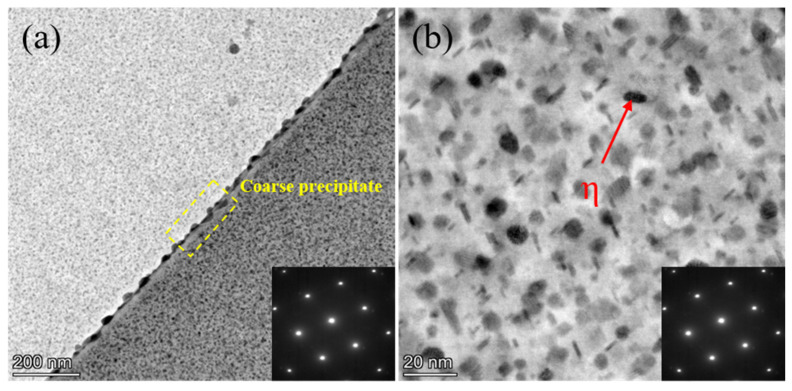
BF-STEM and SAED spots image of SA160-12 h sample ([110]_Al_ axis): (**a**) Grain boundary microstructure; (**b**) Intragranular microstructure.

Figure 5 presents the characteristic microstructure at both the grain boundaries and within the grains of the sample aged at 160 °C for 12 h. As shown in Figure 5a, coarse precipitates are distributed continuously along the grain boundaries. Kawabata et al. [33] reported that such coarse boundary precipitates deplete solute atoms in the adjacent matrix, producing a PFZ on both sides of the boundary. This phenomenon weakens local strengthening and reduces the overall toughness of the alloy. The intragranular precipitates are shown in Figure 5b. Most exhibit spherical or short-rod morphologies. A pronounced increase in the number of η phases is observed, accompanied by a decrease in the number density of η′ precipitates. With prolonged aging, these intragranular precipitates undergo significant coarsening, with average diameters ranging from 6.1 to 10.5 nm. This coarsening alters the precipitate size distribution and may reduce the precipitation-strengthening effect, ultimately degrading the alloy’s mechanical performance.

**Figure 6:**
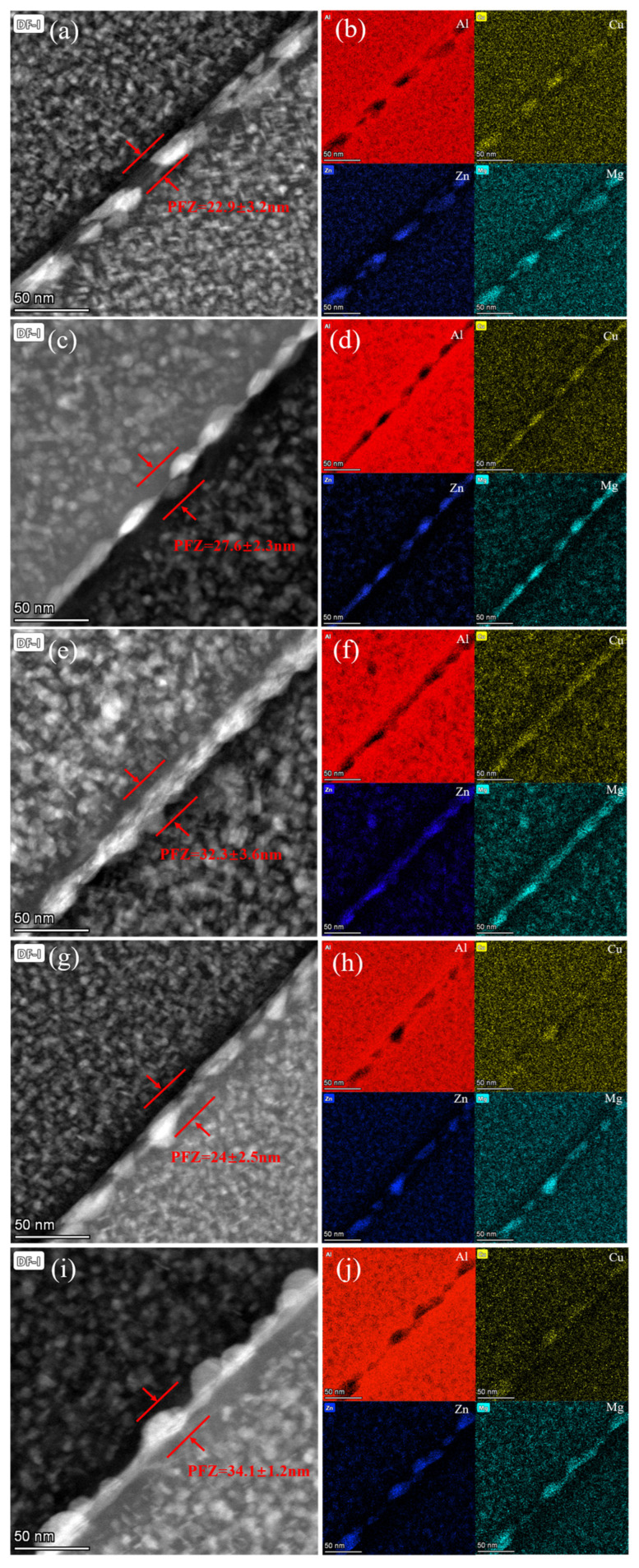
DF-STEM and EDS diagram of boundary precipitates of samples processed with different SA treatments: (**a**,**b**) SA140-4 h; (**c**,**d**) SA160-6 h; (**e**,**f**) SA180-2 h; (**g**,**h**) SA160-2 h; (**i**,**j**) SA160-12 h.

Figure 6 presents bright-field STEM images and energy-dispersive spectroscopy (EDS) maps illustrating the grain boundaries of samples subjected to various secondary aging temperatures. These images clearly show the distribution of fine precipitates in the matrix, such as GPII zones and metastable η′ precipitates. Additionally, larger rod-like precipitates are observed along the grain boundaries. In the SA140-4 h sample, the grain boundary precipitates (GBPs) measured approximately 19.6 nm in size and were discretely arranged in chains along the grain boundaries, and a PFZ with a width of 22.9 nm was also observed (Figure 6a). As the peak-aging temperature increased to 160 °C (SA160-6 h), the continuity of the GBPs was enhanced, resulting in a quasi-continuous distribution, and the PFZ width increased to 27.6 nm (Figure 6c). When the temperature was further increased to 180 °C (SA180-2 h), the grain boundary precipitation was clearly distributed in a continuous manner and significantly coarsened, which led to a PFZ of 32.3 nm (Figure 6e). The SA160-2 h sample exhibited more continuous grain boundary precipitation, with a significantly narrower PFZ (27.6 nm) compared to SA160-6 h, while in the SA160-12 h sample, the precipitates exhibited a more continuous distribution, with significant coarsening, leading to a wider PFZ (34.1 nm) due to the longer aging time (Figure 6i). Moreover, the interfacial precipitates were rich in Zn, Mg, and Cu, suggesting that these precipitates are stable equilibrium η phases [34]. In addition, previous studies employing EDS line-scan analysis have confirmed that, along directions perpendicular to the grain boundary, the concentrations of Zn and Mg decrease markedly within the PFZ while exhibiting pronounced enrichment at the grain boundary itself [35]. This characteristic “depletion–enrichment” pattern confirms that η-phase precipitates at the boundary consume solute from the adjacent matrix, directly driving the formation and widening of the PFZ.

### 3.2. Mechanical Properties

**Figure 7:**
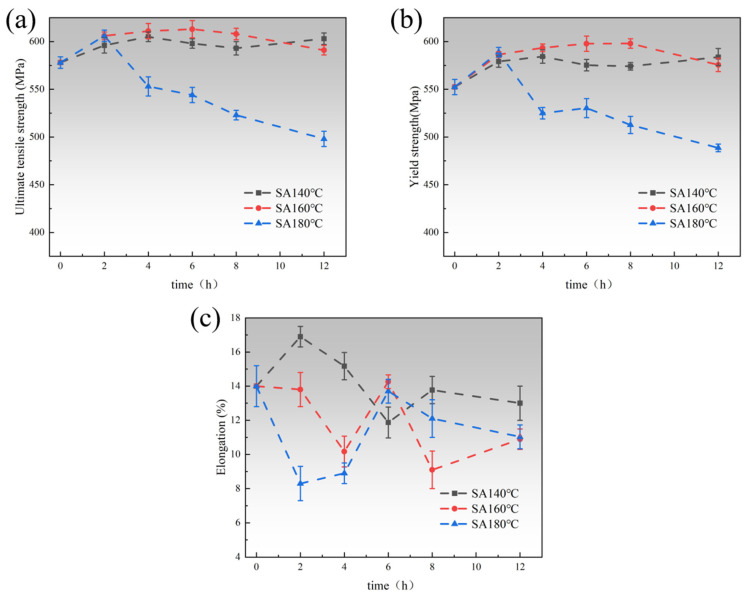
Mechanical property evolution during SA at 140 °C, 160 °C, and 180 °C: (**a**) ultimate tensile strength curves; (**b**) yield strength curves; (**c**) elongation curves.

Figure 7 illustrates the mechanical properties of the samples during secondary aging, including YS, UTS, and EL. It can be observed that specimens subjected to initial pre-aging at 120 °C for 6 h before the second-stage aging exhibited lower strengths, with UTS and YS values of approximately 578 MPa and 552 MPa, respectively. This can be attributed to the shorter pre-aging time, leaving the alloys in an unstable state. During the second-stage aging process, the strength of the alloy at an aging temperature of 140 °C reached the peak-aging plateau after about 4 h (approximately 605 MPa for UTS and 584 MPa for YS) and remained at peak strength for 8 h, with minor fluctuations. As the secondary aging temperature increased to 160 °C, the peak-aging time shortened to 2 h, with minimal fluctuations between 2 h and 8 h, and a peak-aging plateau lasting for 6 h, during which the highest values reached approximately 613 MPa for UTS and 598 MPa for YS. Subsequently, the performance began to decline, showing the over-aging effect. The over-aging effect is more pronounced at 180 °C, reaching its peak in only 2 h (approximately 606 MPa for UTS and 589 MPa for YS), and subsequently, its tensile and yield strengths decrease rapidly as the aging time increases, with no distinct peak-aging plateau observed. The YS and UTS curves indicate that the aging kinetics of the alloy accelerates with increasing secondary aging temperature, leading to the early onset of peak strength and a more pronounced over-aging stage.

During the secondary aging process, the elongation of the samples exhibited an initial decrease, followed by an increase and then a subsequent decrease. For example, the elongation of the SA180 sample decreased significantly after 2 h of aging, which can be attributed to the increased number of precipitates formed during secondary aging, resulting in an enhanced dislocation-pinning effect that adversely affected the alloy’s toughness. However, when the secondary aging time was extended to 6 h, the coarsening and quasi-continuous distribution of the GBPs resulted in a significant increase in the alloy’s elongation. It is noteworthy that the secondary aging of SA160 samples can be primarily divided into two stages: the “0–2 h under-aging zone” and the “2–8 h peak-aging zone.” In the peak-aging zone, the strength remains stable at a relatively high level. In contrast, the SA180 samples exhibit only a brief period of peak strength. The elongation curves indicate that the SA160 samples achieve better elongation at 6 h of secondary aging, reaching 14.26% (Figure 7c), suggesting that an appropriate extension of the secondary aging time can enhance the material’s plasticity and toughness without compromising strength, thereby improving the forming accuracy of the plate. In conclusion, the optimal mechanical properties of the AA7150 alloy were achieved using the 120 °C/6 h + 160 °C/6 h aging process.

## 4. Discussion

### 4.1. Microstructural Evolution

In this study, both the η′ phases and the GPII zones were observed, exerting a critical influence on the strength of Al-Zn-Mg-Cu alloys. To systematically analyze the dynamic evolution of microstructures during secondary aging, a refined characterization of precipitate types and their evolutionary pathways has been intensively conducted [24]. The precipitation kinetics during the secondary aging stage, particularly the metastable-to-equilibrium phase transition, are synergistically regulated by both the thermodynamic driving force and diffusion mechanisms, characterized by multi-scale coupling. Under secondary aging conditions, the interfacial energy-dominated phase coarsening and dislocation-assisted solute migration collectively act to gradually destabilize the GP zones and η′ phases formed during the initial aging process. Consequently, the deconstruction and reorganization of metastable precipitate architectures introduced by pre-aging during secondary aging are primarily governed by long-range solute diffusion driven by vacancy concentration gradients and by the reduction in phase transition barriers induced by the relaxation of lattice strain [36].

**Figure 8:**
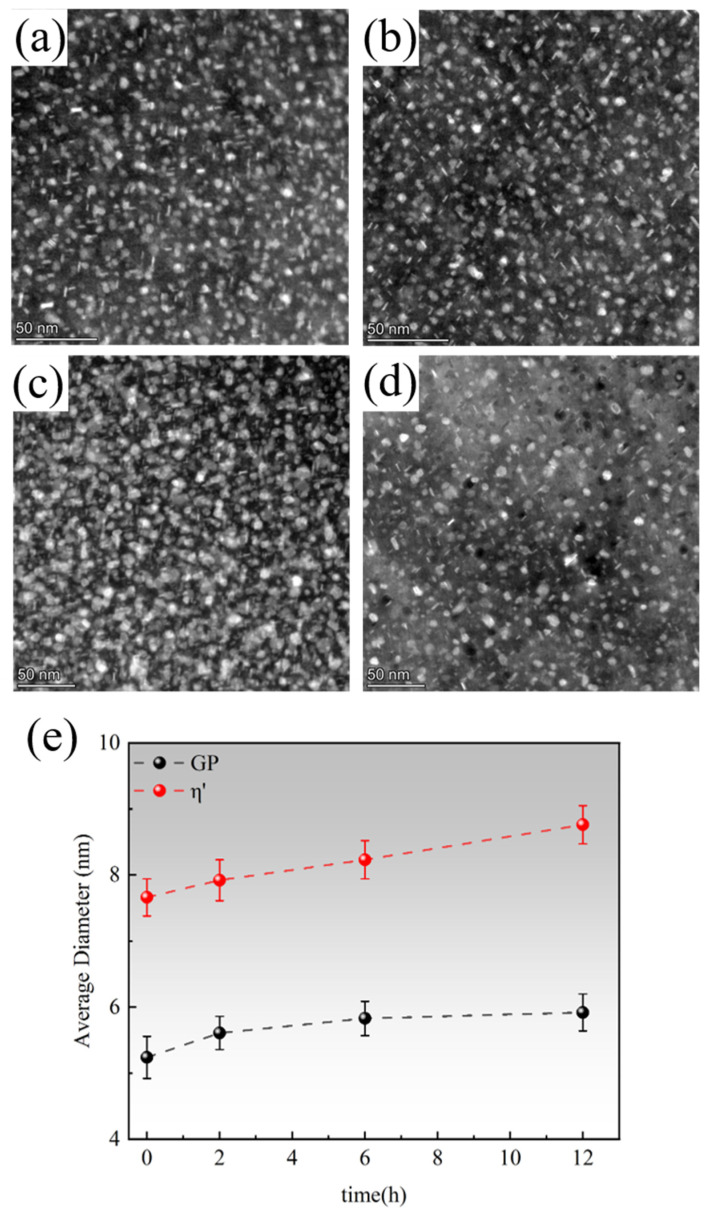
STEM-DF images showing the microstructure of the pre-staged sample and its secondary aging at 160 °C: (**a**) pre-staged sample; (**b**) secondary aging for 2 h; (**c**) secondary aging for 6 h; (**d**) secondary aging for 12 h; (**e**) average diameter of the secondary aging sample.

Figure 8 illustrates the evolution of precipitate size during the secondary aging process of the sample following a 6 h pre-aging treatment at 120 °C. After pre-aging, the predominantly metastable GPII zone and η′ phase were formed in the samples; these two precipitates are generally considered the main strengthening phases in Al-Zn-Mg-Cu alloys [37]. During secondary aging, the solute atoms in the matrix begin to diffuse and redistribute under high-temperature excitation at 160 °C. This results in solutes released from the partial dissolution of smaller GPII zones being captured by adjacent larger zones, while localized merging or co-growth effects may occur between adjacent GPII zones, leading to a faster growth rate of the GPII zone size (Figure 8a,b). In the early stage of secondary aging (2–6 h), the η′ phase undergoes coarsening primarily through the Ostwald ripening mechanism: small-sized η′ phases gradually dissolve, while large-sized η′ phases grow. Simultaneously, the localized solute supersaturation regions facilitate the initiation of η′ phase precipitation through a heterogeneous nucleation mechanism [38]. At this stage, although the average length of the η′ phase continued to increase, its growth rate leveled off (Figure 8e). In contrast, the GPII zone began to dissolve rapidly (Figure 8a–c), leading to a significant decrease in its number, with its dissolution rate also showing a decreasing trend. After 6 h of secondary aging, the size growth of the GPII zone gradually stabilizes, and the GPII zone in the matrix is essentially replaced by the metastable η′ phase. During the decomposition of the GPII zone, the released Zn/Mg atoms diffuse through the dislocation network, providing a driving force for the further growth of the η′ phase (Figure 8c). Although the growth rate of the precipitates slowed during secondary aging, their overall size continued to increase over time (Figure 8e). As the aging time extends to 12 h, the prolonged aging provides sufficient diffusion time for the solute atoms, allowing the metastable η′ phase to gradually transform into the thermodynamically more stable equilibrium η phase. The transformation process is driven by the minimization of the system’s free energy and is kinetically accelerated by vacancy-assisted solute diffusion and interfacial strain relaxation. The η phase usually nucleates preferentially at the η′-phase-matrix interface or grain boundary defects, and coarsening occurs through the depletion of Zn/Mg atoms in the η′ phase (Figure 8d). This results in a reduction in the number and size of the η′ phase, with the size distribution becoming polarized, leading to the formation of an over-aged microstructure dominated by the incoherent η phase.

Grain boundaries, acting as effective enrichment zones for solute atoms and vacancies, often serve as locations for the aggregation of precipitated phases [39]. In aluminum alloys, the formation of the equilibrium η phase at grain boundaries and its coarsening process are typically accompanied by the formation of a PFZ along the grain boundaries [40]. This phenomenon negatively impacts the mechanical properties of aluminum alloys, primarily manifesting as a decrease in strength due to the weakening of the precipitation hardening effect within the PFZ region [41]. As shown in Figure 6, during the secondary aging process at 160 °C, prolonged aging exacerbates the diffusion of solute atoms along the grain boundaries, promoting the precipitation of the coarse equilibrium η phase. This results in a significant increase in the PFZ width and the average size of the GBPs. The formation of coarse precipitates causes significant depletion of solute atoms near the grain boundaries, thereby reducing the local solute atom concentration in this region.

### 4.2. Microstructure and Properties

To elucidate the dominant effect of secondary aging on the mechanical behavior of AA7150 aluminum alloy, this study focuses on the coupling relationship between the dynamic evolution of the precipitated phases and the strengthening mechanism. According to references [42,43], secondary aging significantly governs the precipitation strengthening effect by modulating the size, volume fraction, and distribution characteristics of the precipitated phases. Precipitation strengthening is the primary factor in improving the mechanical properties of Al-Zn-Mg-Cu alloys [44,45]. The precipitation strengthening mechanism was determined by comparing the strength increments associated with dislocation shear and Orowan bypass mechanisms. An operational mechanism was developed based on smaller strength increments [46]. The Orowan bypass mechanism targets non-shearable precipitates, such as the roughened η phase, where dislocations form Orowan rings by bypassing. Its strength increment can be expressed by the following equation [47]:

(1) $$\Delta \sigma_{Or}=M\frac{0.4Gb}{\pi \sqrt{1-\nu }}\cdot \frac{\ln\left(2\sqrt{\frac{2}{3}r}/b\right)}{\lambda_{p}}$$

This equation describes the Orowan dislocation bypassing mechanism and incorporates several parameters including Poisson’s ratio (ν), which is typically about 0.33 for the AA7150 alloy. Additionally, the edge-to-edge inter-precipitate distance ($\lambda_{p}$) is taken into account, where $\lambda_{p}$ is expressed as $\lambda_{p}=2\sqrt{\frac{2}{3}}\;r\left(\sqrt{\frac{\pi }{4f}}-1\right)$, where r and f denote the average radius and volume fraction of the precipitates, respectively.

The strength of a material is primarily influenced by fine intracrystalline precipitates and dispersed phases. When the GBPs are relatively fine and predominantly consist of metastable GPII regions and fine η′ precipitates, dislocations can easily shear these precipitates. Therefore, the strengthening effect is as follows [48]:

(2) $$\Delta \tau_{\mathrm{cut}}=\alpha f^{1/2}r^{1/2}$$

In the above equation, α represents a constant, the interfacial reinforcement factor, f represents the volume fraction of the precipitate, and r corresponds to the radius of the precipitate.

Therefore, for larger precipitates, the strength of Al-Zn-Mg alloys exhibits a negative correlation with the radius of the precipitates and a positive correlation with the volume fraction. In contrast, for fine precipitates, the strength is positively correlated with both the radius and the volume fraction of the precipitates.

Figure 3a illustrates that the primary precipitates in the SA140-4 h samples at the peak-aging plateau are the η′ phase, along with a small number of GPII zones. These precipitates are small in size, high in number, and maintain an incoherent or semi-coherent relationship with the matrix, causing dislocations to pass through them predominantly via shear mechanisms, thus yielding a limited strengthening effect. However, for the SA160-6 h sample at a higher temperature, the precipitate phase evolved into a larger number of more stable semi-coherent η′ phases, and the GPII zone nearly disappeared (Figure 3b). The secondary aging condition of SA160-6 h promoted the precipitation of additional solute atoms, resulting in a higher overall number of precipitates. Although the size of the individual precipitates increased, the higher density and more homogeneous distribution likely provided more dislocation pinning points. Furthermore, the semi-coherent lattice relationship enhanced the binding of the precipitate phase, further hindering dislocation motion, and the coarsening of the η′ phase enhanced the Orowan bypass mechanism. As a result, the SA160-6 h sample exhibits greater strength compared to the SA140-4 h sample (Figure 7b).

Compared with the SA140-4 h and SA160-6 h samples, the SA180-2 h sample exhibited a small amount of non-uniform coarse η phase (Figure 3c). The high-temperature, short-duration aging led to the rapid nucleation of precipitated phases, but they did not distribute uniformly in time. Additionally, the merging of precipitates in some regions reduced the overall number of precipitates that effectively hindered dislocations. The transformation of the η′ phase into the η phase weakened both the shear-strengthening and Orowan bypass-strengthening effects. As a result, the strength of the SA180-2 h sample is lower compared to that of the SA160-6 h sample (Figure 7b).

**Figure 9:**
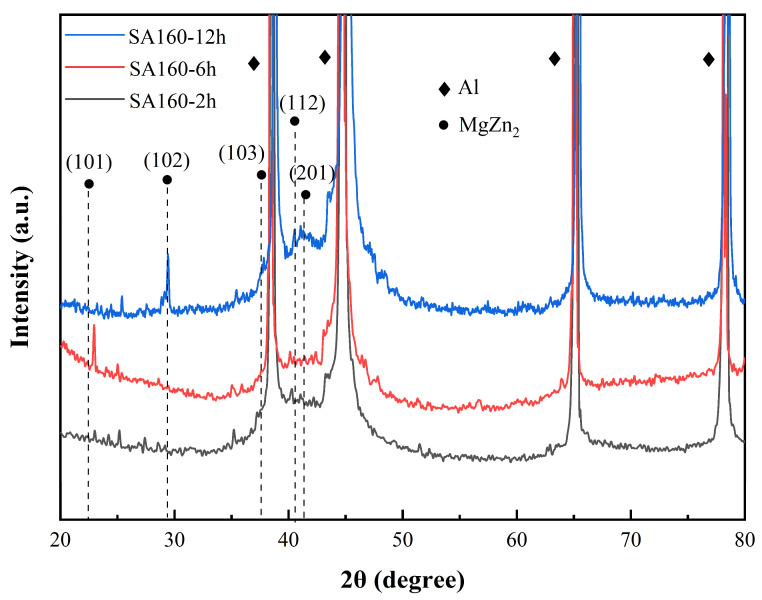
XRD patterns of Al-Zn-Mg-Cu alloy after treatment with different secondary aging times at 160 °C.

Figure 9 illustrates the evolution of XRD patterns for Al-Zn-Mg-Cu alloys subjected to different treatment times at a secondary aging temperature of 160 °C. The curves reveal that the individual characteristic peaks corresponding to the metastable η′ and equilibrium η phases are weaker for the SA160-2 h sample, suggesting that the total volume fraction of precipitated phases in the alloy remains low. This phenomenon can be attributed to the dual constraints of nucleation barriers and diffusion kinetics, where the shorter secondary aging time does not allow sufficient time for the precipitates to form nuclei and grow. The insufficient precipitation results in a less pronounced aging strengthening effect, leading to lower alloy strengths. As the aging time was extended to 6 h, the intensity of the characteristic peak at 2θ = 23° in the XRD pattern significantly increased, with the peak shifting to the right of the standard η phase (101) diffraction. TEM analysis revealed that the content of GP zones had diminished considerably, and the sample was primarily composed of η and η′ phases. The diffraction pattern corresponding to 2θ = 23° deviated from the (101) diffraction of the η phase, indicating the presence of η′ phase, which demonstrates the dense precipitation behavior of the metastable η′ phase. This precipitation kinetics aligns with the classical nucleation-growth theory, where prolonged aging treatment provides solute atoms with sufficient diffusion activation energy, facilitating the transformation of GP zones into the η′ phase and enhancing aging strengthening. Further extending the aging time to 12 h resulted in a slight weakening of the peak near 2θ = 23°, while peaks at 2θ = 29° and 37° exhibited notable intensification and became sharper, corresponding to the η phase (102) and (103) plane diffractions, respectively. Additionally, it was observed that the peaks in the 2θ range of 40° to 43° exhibited broadening in all three aging times, which primarily corresponded to the diffractions of GPII and η′ phases. In this range, at 12 h, the diffraction peaks of the η phase (112) and (201) planes were significantly enhanced. Lattice constants were refined using the Jade software (version 9.02), yielding values of a = b = 5.15 Å and c = 8.48 Å, consistent with previous studies [49,50]. This indicates that the transformation from the metastable to the equilibrium phase entered the Ostwald ripening stage, with an increased proportion of the η′ phase. This is due to the extended aging time at a constant temperature, which creates favorable conditions for the growth and coarsening of precipitates. The transformation follows the precipitation sequence, where smaller η′ particles dissolve, and solute atoms are transported to larger η phase particles via bulk diffusion, causing precipitate coarsening and loss of interfacial cohesion. This phase transition is accompanied by a reduction in strain field strengthening and a weakening of the dislocation cutting mechanism, leading to a decrease in strength. Consequently, the best strength properties were observed in the SA160-6 h samples, as shown in Section 3.2, with a UTS of 613 MPa, a YS of 598 MPa, and an elongation of 14.26%. Compared with other peak-aging treatments (e.g., SA140-4 h and SA180-2 h), the SA160-6 h condition achieves a superior balance between strength and ductility. Moreover, relative to conventional T6 processing or retrogression and re-aging (RRA, 120 °C/24 h + 190 °C/0.5 h + 120 °C/24 h) [51], the SA160-6 h delivers comparable mechanical properties (T651 condition [52], UTS ~600 MPa, EL ~15.6%; RRA condition [51], UTS ~600 MPa, EL ~10%) while significantly reducing the overall processing time, thereby demonstrating both superior efficiency and performance advantages.

## 5. Conclusions

This study systematically investigates the effect of secondary aging on the precipitation behavior and mechanical properties of Al-Zn-Mg-Cu alloys. A two-step aging strategy (120 °C/6 h pre-aging + 160 °C/6 h SA) is proposed and validated. Compared with conventional T6 and RRA treatments, this approach not only significantly reduces the overall processing time but also avoids the complex operations and stability risks associated with the rapid high-temperature regression step in RRA, ultimately achieving an excellent balance between strength and ductility. The following conclusions are drawn:

(1) After the two-step aging treatment, the precipitates primarily consisted of the metastable η′ phase and the equilibrium η phase, with a relatively uniform distribution. Precipitate strengthening is the primary mechanism for strengthening the alloy, and a precipitation-free zone (PFZ) is clearly observed at the grain boundaries. Additionally, it was found that increasing the secondary aging temperature shortened the time required for the alloy to reach the peak-aging plateau.
(2) Under the regulation of secondary aging temperatures (140 °C, 160 °C, 180 °C), the samples subjected to peak-aging at 160 °C exhibited the optimal overall performance (tensile strength of 613 MPa, yield strength of 589 MPa, and elongation of 14.26%). Additionally, it was observed that with the extension of aging time, the longest duration of the peak plateau period was 6 h at 160 °C, and elongation increased slightly during this period. This indicates that appropriately extending the secondary aging time at this temperature can enhance the plasticity and toughness of the material without a loss in strength.
(3) A relatively short secondary aging time can result in the metastable η′ strengthening phase not having enough time to nucleate and grow, leading to a less pronounced aging strengthening effect. On the other hand, excessive prolongation of the secondary aging time results in the formation of a large number of equilibrium η phases. The larger size of the equilibrium η phase not only fails to strengthen the alloy but also consumes a large amount of zinc and magnesium, restricting the precipitation of the η′ phase during aging. Additionally, the widening of the PFZ around the coarse equilibrium η phases further reduces the mechanical properties of the alloy.

## Figures and Tables

**Table 1:** The main chemical composition of alloy 7150 (wt.%) [26].

Zn	Mg	Cu	Mn	Fe	Si	Ni	Cr	Ti	Al
6.1	2.19	2.19	<0.01	0.083	0.048	<0.01	0.16	0.025	Bal.

## Data Availability

The datasets presented in this article are not readily available because the data are subject to technical/time limitations. Requests to access the datasets should be directed to corresponding author.

## References

[1] Dong Y., Ye L., Tang J., Liu X., Sun Q. (2020). The effects of temperature on the creep-aging behavior and mechanical properties of AA2050-T34 alloy. Mater. Sci. Eng. A.

[2] Khamneh M.E., Askari-Paykani M., Shahverdi H., Hadavi S.M.M., Emami M. (2016). Optimization of spring-back in creep age forming process of 7075 Al-Alclad alloy using D-optimal design of experiment method. Measurement.

[3] Lam A.C.L., Shi Z., Lin J., Huang X. (2015). Influences of residual stresses and initial distortion on springback prediction of 7B04-T651 aluminium plates in creep-age forming. Int. J. Mech. Sci..

[4] Dorward R.C., Pritchett T.R. (1988). Advanced aluminium alloys for aircraft and aerospace applications. Mater. Des..

[5] Davis J.R. (1993). Aluminum and Aluminum Alloys.

[6] Österreicher J.A., Grabner F., Tunes M.A., Coradini D.S.R., Pogatscher S., Schlög C.M. (2021). Two step–ageing of 7xxx series alloys with an intermediate warm-forming step. J. Mater. Res. Technol..

[7] Wolverton C. (2001). Crystal structure and stability of complex precipitate phases in Al–Cu–Mg–(Si) and Al–Zn–Mg alloys. Acta Mater..

[8] Li J.F., Peng Z., Li C.X., Jia Z.Q., Chen W.J., Zheng Z.Q. (2008). Mechanical properties, corrosion behaviors and microstructures of 7075 aluminium alloy with various aging treatments. Trans. Nonferrous Met. Soc. China.

[9] Österreicher J.A., Tunes M.A., Grabner F., Arnoldt A., Kremmer T., Pogatscher S., Schlögl C.M. (2020). Warm-forming of pre-aged Al-Zn-Mg-Cu alloy sheet. Mater. Des..

[10] Zhang N., Lei C., Tang H., Wang Q. (2022). Double-step aging treatment of high strength Al-5 Mg-3Zn-1Cu (wt%) cast alloy. Mater. Lett..

[11] Chen J., Cheng X., Ding L., Weng Y., Yin J., Yao H., Yu H. (2022). Effect of multi-stage aging on the precipitation strengthening and mechanical properties for an Al-Mg-Si-Ag alloy. Mater. Charact..

[12] Guo J., Su R., Li G., Qu Y. (2024). Effect of secondary aging on microstructure and properties of cast Al–Cu–Mg–Ag alloy. Int. J. Met..

[13] Zhao W., Wang L., Ren Y., He B., Han S., Xu P. (2023). Effect of precipitates evolution on mechanical properties of Al 7050 alloy during secondary aging. Mater. Res. Express.

[14] Lee Y.S., Koh D.H., Kim H.W., Ahn Y.S. (2018). Improved bake-hardening response of Al-Zn-Mg-Cu alloy through pre-aging treatment. Scr. Mater..

[15] Stemper L., Tunes M.A., Dumitraschkewitz P., Mendez-Martin F., Tosone R., Marchand D., Curtin W.A., Uggowitzer P.J., Pogatscher S. (2021). Giant hardening response in AlMgZn (Cu) alloys. Acta Mater..

[16] Lumley R.N., Polmear I.J., Morton A.J. (2004). Temper developments using secondary ageing. Mater. Forum.

[17] Sun Y., Jiang F., Zhang H., Su J., Yuan W. (2016). Residual stress relief in Al–Zn–Mg–Cu alloy by a new multistage interrupted artificial aging treatment. Mater. Des..

[18] Yan S., Wang R., Peng C., Cai Z., Peng X., Li Z., Wu Z. (2025). Enhancing the strength and ductility of rapidly-solidified 2196 Al-Li alloy through multi-stage ageing treatments. Vacuum.

[19] Li K., Liu K., Su R., Li G., Qu Y. (2025). The Effect of Multi-Stage Aging Treatment on the Microstructure and Properties of 7075 Aluminum Alloy. J. Alloys Compd..

[20] Clinch M.R., Daval R., Harris S.J., Hepples W., Holroyd N.J.H., Lawday M.J., Noble B. (2004). A Microstructural Engineering-Based Approach to 7 xxx Series Alloy Optimisation. Mater. Forum.

[21] Wang S., Luo B., Bai Z., He C., Jiang G. (2024). Effect of pre-ageing on nucleating of GP zones and precipitation, strength and stress corrosion properties of 7N01 alloy. J. Alloys Compd..

[22] Li Z.H., Xiong B., Zhang Y., Zhu B., Wang F., Liu H. (2009). Investigation on strength, toughness and microstructure of an Al–Zn–Mg–Cu alloy pre-stretched thick plates in various ageing tempers. J. Mater. Process. Technol..

[23] Li Z.H., Xiong B., Zhang Y., Zhu B., Wang F., Liu H. (2007). Effects of the two-step ageing treatment on the microstructure and properties of 7B04 alloy pre-stretched thick plates. Rare Met..

[24] Azarniya A., Taheri A.K., Taheri K.K. (2019). Recent advances in ageing of 7xxx series aluminum alloys: A physical metallurgy perspective. J. Alloys Compd..

[25] Li C., Wei H.B., Xu X.J., Zhou Q., Han M.N., Sha S.H. (2024). Microstructure and mechanical properties of a highly alloyed Al–Zn–Mg–Cu–Zr alloy under T6I4 and T6R6I4 agings. Met. Mater. Int..

[26] Zeng Q., Feng S., Chen F., Wang D., Zhan L., Yang Y., Tang L., Liu C., Yan D. (2024). Improving creep-aging behavior and mechanical properties of AA7150 alloy via pre-aging. Mater. Sci. Eng. A.

[27] Yang W., Ji S., Wang M., Li Z. (2014). Precipitation behaviour of Al–Zn–Mg–Cu alloy and diffraction analysis from η′ precipitates in four variants. J. Alloys Compd..

[28] Sha G., Cerezo A. (2004). Early-stage precipitation in Al–Zn–Mg–Cu alloy (7050). Acta Mater..

[29] Cao F., Qin Z. (2021). Effect of two-stage aging on hardness and electrical conductivity of as-extruded 7075 aAluminum Alloy. J. Phys. Conf. Ser..

[30] Zhao J., Bai S., Huang T., Wang J., Xie H., Zeng D., Luo L. (2021). Effect of various aging treatment on thermal stability of a novel Al-Zn-Mg-Cu alloy for oil drilling. Mater. Sci. Eng. A.

[31] Zou Y., Cao L., Wu X., Tang S., Guo M. (2023). Synergetic effect of natural ageing and pre-stretching on the ageing behavior in T′/η′ phase-strengthened Al-Zn-Mg-Cu alloys. J. Mater. Sci. Technol..

[32] Chen J., Zhen L., Yang S., Shao W., Dai S. (2009). Investigation of precipitation behavior and related hardening in AA 7055 aluminum alloy. Mater. Sci. Eng. A.

[33] Kawabata T., Izumi O. (1976). Ductile fracture in the interior of precipitate free zone in an Al-6.0% Zn-2.6% Mg alloy. Acta Metall..

[34] Liu J., Du Z., Su J., Tang J., Jiang F., Fu D., Teng J., Zhang H. (2023). Effect of quenching residual stress on precipitation behaviour of 7085 aluminium alloy. J. Mater. Sci. Technol..

[35] Wu M., Xiao D., Yuan S., Huang Y., Li Z., Yin X., Wang J., Huang L., Liu W. (2024). Healing the high-temperature-retrogression-caused wide precipitation-free zones in Al-Zn-Mg-Cu alloy via strain-aging induced precipitates. Mater. Sci. Eng. A.

[36] Marceau R.K.W., Sha G., Lumley R.N., Ringer S.P. (2010). Evolution of solute clustering in Al–Cu–Mg alloys during secondary ageing. Acta Mater..

[37] Yu X.W., Chen J.H., Li J.Y., Wu C.L., Yang X.B. (2019). Effect of pre-deformation on quench-induced inhomogeneity of microstructure and hardness in 7050 aluminum alloy. Mater. Charact..

[38] Yu Z., Li H., Cai P., Fu X., Feng Z., Zhang L., Wang J., Xiao N. (2023). Effect of aging route on the precipitation behavior and thermal stability of Al–Cu–Mg–Ag alloy. J. Mater. Res. Technol..

[39] Zheng Y., Li C., Liu S., Deng Y., Zhang X. (2014). Effect of homogenization time on quench sensitivity of 7085 aluminum alloy. Trans. Nonferrous Met. Soc. China.

[40] Chobaut N., Carron D., Arsène S., Schloth P., Drezet J.M. (2015). Quench induced residual stress prediction in heat treatable 7xxx aluminium alloy thick plates using Gleeble interrupted quench tests. J. Mater. Process. Technol..

[41] Steele D., Evans D., Nolan P., Lloyd D.J. (2007). Quantification of grain boundary precipitation and the influence of quench rate in 6XXX aluminum alloys. Mater. Charact..

[42] Afifi M.A., Wang Y.C., Langdon T.G. (2020). Effect of dynamic plastic deformation on the microstructure and mechanical properties of an Al–Zn–Mg alloy. Mater. Sci. Eng. A.

[43] Bignon M., Ma Z., Robson J.D., Shanthraj P. (2023). Interactions between plastic deformation and precipitation in Aluminium alloys: A crystal plasticity model. Acta Mater..

[44] Ye J., Pan Q., Liu B., Hu Q., Qu L., Wang W., Wang X. (2023). Effects of co-addition of minor Sc and Zr on aging precipitates and mechanical properties of Al–Zn–Mg–Cu alloys. J. Mater. Res. Technol..

[45] Lee S.H., Jung J.G., Baik S.I., Seidman D.N., Kim M.S., Lee Y.K., Euh K. (2021). Precipitation strengthening in naturally aged Al–Zn–Mg–Cu alloy. Mater. Sci. Eng. A.

[46] Wen H., Topping T.D., Isheim D., Seidman D.N., Lavernia E.J. (2013). Strengthening mechanisms in a high-strength bulk nanostructured Cu–Zn–Al alloy processed via cryomilling and spark plasma sintering. Acta Mater..

[47] Heo J.G., Lee Y.S., Kim M.S., Kim H.W., Kim Y.D. (2019). Effect of pre-aging treatment on bake-hardenability of Al-8.0 Zn-2.5 Mg-2.0 Cu alloy sheet fabricated by twin-roll casting process. Korean J. Met. Mater..

[48] Lee S.H., Jung J.G., Baik S.I., Park S.H., Kim M.S., Lee Y.K., Euh K. (2021). Effects of Ti addition on the microstructure and mechanical properties of Al–Zn–Mg–Cu–Zr alloy. Mater. Sci. Eng. A.

[49] Liu S., Hou H., Shao W., Yang J., Wang Z., Yang Q., LLorca J. (2024). Revisiting the precipitation mechanisms of Guinier-Preston zones, η′, and η precipitates in Al-Zn-Mg alloys. Acta Mater..

[50] Xiang K., Ding L., Jia Z., Yang X., Liu Q., Hao Z. (2022). Phase transition induced by synchroshear in Al-Zn-Mg-Cu alloy. Scr. Mater..

[51] Chen K., Zhan L., Xu Y., Ma B., Zeng Q., Luo S. (2022). Optimizing strength and ductility in 7150 Al alloys via rapid electropulsing cyclic heat treatment. J. Alloys Compd..

[52] Chen K., Zhan L., Yu W. (2021). Rapidly modifying microstructure and mechanical properties of AA7150 Al alloy processed with electropulsing treatment. J. Mater. Sci. Technol..

